# Widespread Recommendations Can Change Our Habits of Hand-Washing and Physical Distance During the COVID-19 Pandemic

**DOI:** 10.32872/cpe.3061

**Published:** 2021-03-10

**Authors:** Stefanie C. Biehl, Melissa Schmidmeier, Theresa F. Wechsler, Leon O. H. Kroczek, Andreas Mühlberger

**Affiliations:** a Department of Clinical Psychology and Psychotherapy, University of Regensburg, Regensburg, Germany; Philipps-University of Marburg, Marburg, Germany

**Keywords:** COVID-19 pandemic, everyday habits, hand-washing, physical distancing

## Abstract

**Background:**

Habits and behaviors in everyday life currently need to be modified as quickly as possible due to the COVID-19 pandemic. Two of the most effective tools to prevent infection seem to be regular and thorough hand-washing and physical distancing during interpersonal interactions.

**Method:**

Two hundred and eighty-four participants completed a short survey to investigate how previous habits regarding hand-washing and physical distancing have changed in the general population as a function of the current pandemic and the thereby increased information and constant recommendations regarding these behaviors.

**Results:**

Participants aged 51 and older reported a greater change in everyday hand-washing behavior than younger participants. In addition, participants aged 31 and older selected significantly greater distances to have a conversation than younger participants. However, that was not the case if participants had to actively stop their conversational partner from approaching.

**Conclusion:**

Participants aged 51 years and older seem to be well aware of their at-risk status during the current pandemic and might therefore be willing to change their behavior more strongly than younger survey participants. Nevertheless, they seem to struggle with enforcing the current rules towards others. The group aged between 31 and 50 years, however, reports a comparable level of fear, but no corresponding change in hand-washing behavior. Future surveys should try to provide more insight into why this might be the case.

## Abstract (highlights)

- Habits and behaviors need to be modified quickly during the current COVID-19 pandemic.
- Participants aged 51 years and older seem to be willing to change their behavior more strongly.
- However, they seem to struggle with enforcing some of the current rules towards others.

The current COVID-19 pandemic forces us to change our everyday lives and associated habits as quickly as possible. Regular thorough hand-washing and physical distancing have been recommended as two of the most effective tools to prevent infection (Bundeszentrale für gesundheitliche Aufklärung, 2020). Habits regulating these behaviors, however, are triggered by similar contextual circumstances, can be implemented using minimal resources, and can be used to predict future behavior in a similar situation (for a review see Ouellette & Wood, 1998). Habitual behavior thus needs to be modified by consciously inhibiting previously established habitual behavior and implementing alternative responses (for a review see Gardner, 2015). Social psychological models furthermore suggest that social behavior is not only driven by a reflective system based on consequences and probabilities, but also by an impulsive system based on spreading activation (Strack & Deutsch, 2004), which can cause fear to at least co-determine behavior.

Regular thorough hand-washing is already recommended during periods of increased probability of infections to prevent the spreading of infectious diseases like influenza (Bundeszentrale für gesundheitliche Aufklärung, 2018). Previous population-based research, however, does not show a clear reduction in influenza transmission (Simmerman et al., 2011) or acute respiratory tract infections (Merk, Kühlmann-Berenzon, Linde, & Nyrén, 2014) as a function of self-reported hand-washing. Of note, the latter investigation suggested a protective effect for health-care workers, leading the authors to conclude that the knowledge regarding adequate hand-washing might be insufficient in the general population.

We implemented a short survey to investigate how previous habits regarding hand-washing were changed in the general population as a function of the current pandemic and the thereby increased information and constant recommendations regarding adequate hand-washing. We also assessed whether the general public is aware of and able to follow further recommendations, particularly with regard to physical distancing in interpersonal situations.

## Method

### Assessment

Data were collected for the duration of twelve days, starting on the day of the implementation of movement restrictions in Bavaria (March 21^st^, 2020) and ending on April 1^st^. The questionnaire was implemented via EvaSys (Electric Paper Evaluationssysteme GmbH, Lueneburg, Germany), an online questionnaire tool operated by the University of Regensburg. It consisted of seven questions assessing the frequency of hand-washing in different situations as well as possible changes since the outbreak of the corona virus SARS-CoV-2. Situations were chosen to cover a range of everyday situations, in which hand-washing is recommended (before eating, after entering your flat/house, after blowing your nose, after coughing/sneezing in your hand, after touching another person not living in the same household, after touching an object that is also touched by other people) as well as a baseline item (after using the bathroom). Participants were asked to report both the frequency of and the change in hand-washing in these situations on a five-point scale (“0 = never” to “4 = always” and “0 = unchanged” to “4 = very much more”, respectively). Data were aggregated to form mean scores across situations with self-reference (before eating, after entering your flat/house, after blowing one’s nose, after coughing/sneezing in your hand) and with other-reference (after touching another person/an object touched by other people), both for frequency and change since the outbreak of the virus.

**Figure 1:**
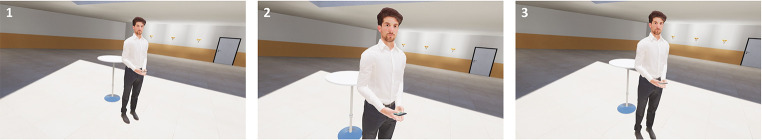
Virtual Reality Pictures Used in the Assessment of Physical Distancing *Note.* Standardized pictures from virtual reality with an agent at the distances of 1.5m (1), 1m (2), and 2m (3) taken from an experimental VR-paradigm, joint project OPTAPEB. ©VTplus.

In addition, the questionnaire assessed the use of soap/disinfectant, the adherence to further recommendations to avoid infection (not touching one’s face and physical distancing), the subjective importance of following the recommendations regarding hand-washing, and the attention to observing adequate physical distance during interactions. Participants were also asked to select interpersonal distances where they a) were currently most comfortable with (passive distancing) and b) would stop someone else from approaching (active distancing) from one of three standardized virtual reality pictures showing an agent at the distances of 1m, 1.5m, and 2m (see Figure 1), which were taken as still frames from a Virtual Reality scenario (VTplus GmbH, Würzburg, Germany). Furthermore, participants’ fear of COVID-19 for themselves and for relatives as well as the incidence of pathological hand-washing as occurring in obsessive compulsive disorder (OCD; i.e. washing one’s hands more frequently and longer than necessary) were assessed.

Participants were informed beforehand that participation in the survey was entirely voluntary and that they could end the survey at any time, in which case no data were transmitted. To comply with current regulations of data protection and to ensure de facto anonymity, age was only collected in the form of age ranges (5 years per range except for 18 to 21 years). Care was furthermore taken to keep the survey as short as possible and to not include questionnaires that might cause distress in survey participants (e.g. assessing mental health problems). All participants gave their informed consent to participate in the survey.

A link to access the questionnaire was distributed via personal contacts, social media, university mailing lists, and a press release on the university’s home page.

### Participants

A total of 284 adults (205 women) between 18 and 75 years of age participated in the survey. While participants’ place of residence was not obtained to ensure anonymity, 93.7% of the sample (266 participants) reported movement restrictions at their place of residence when taking the survey. As this was not the case for 62.5% of the German federal states at the time of data collection (Steinmetz, Batzdorfer, & Bosnjak, 2020), it is likely that most participants lived in Bavaria at the time of the survey. Overall, 72.2% of participants were aged 40 years or younger, with the largest percentage of participants (30.6%) in the 21 to 25 years age group. To facilitate analyses, participants were assigned to one of the age groups: “young age” (YA, 18-30 years of age; 150 participants); “middle age” (MA, 31-50 years of age; 86 participants), and “best/older age” (OA, >50 years of age; 48 participants). The category “best/older age” was chosen to include all participants with a theoretically increased risk for severe or critical course of COVID-19, as the Robert Koch-Institute lists older people as having a steadily increased risk for a severe course of the disease, starting at age 50 to 60. (Robert Koch-Institut, 2021). There was a trend for a greater proportion of women in the YA group, χ^2^(2) = 5.2, *p* = .074; see Table 1 for descriptive data. Most participants (78.9%) reported high-school level education (*Abitur*), with 39.4% of the sample currently attending university.

**Table 1:** Descriptive Data for the Younger Age (YA), Middle Age (MA), and Best/Older Age (OA) Groups

Behavior	YA		MA		OA	
	*M*	*SD*	*M*	*SD*	*M*	*SD*
Use of soap	3.8	0.4	3.7	0.6	3.9	0.5
Use of disinfectant	1.4	1.1	1.2	1.0	1.3	1.1
Trying not to touch one’s face	2.6	1.0	2.6	1.0	2.7	1.1
Average number of people met per day^a^	3.2	10.6	2.1	5.9	2.5	3.8
Pathological hand-washing	1.4	1.1	1.1	1.1	1.4	1.3
Importance of observing hand-washing	3.6	0.7	3.6	0.6	3.9	0.4
Attention to physical distance	3.4	0.7	3.6	0.6	3.8	0.5
	*n*	*n* female	*n*	*n* female	*n*	*n* female
	150	116	86	59	48	30

*Note.* Means and standard deviations for use of soap and disinfectant, trying not to touch one’s face, average amount of people met per day, pathological hand-washing, subjective importance of hand-washing, and attention to physical distancing (on 5-point scales starting at 0) for the three groups.

^a^not belonging to one’s household.

## Results

Descriptive data showed a mean frequency of hand-washing across all age groups and situations slightly below the “3 = often” scale point (*M* = 2.7, *SD* = 0.8) on a five-point scale (“0 = never” to “4 = always”), and a mean change in hand-washing frequency slightly above the “2 = somewhat changed” scale point (*M* = 2.3, *SD* = 1.0), also on a five-point scale (“0 = unchanged” to “4 = very much more”).

**Figure 2:**
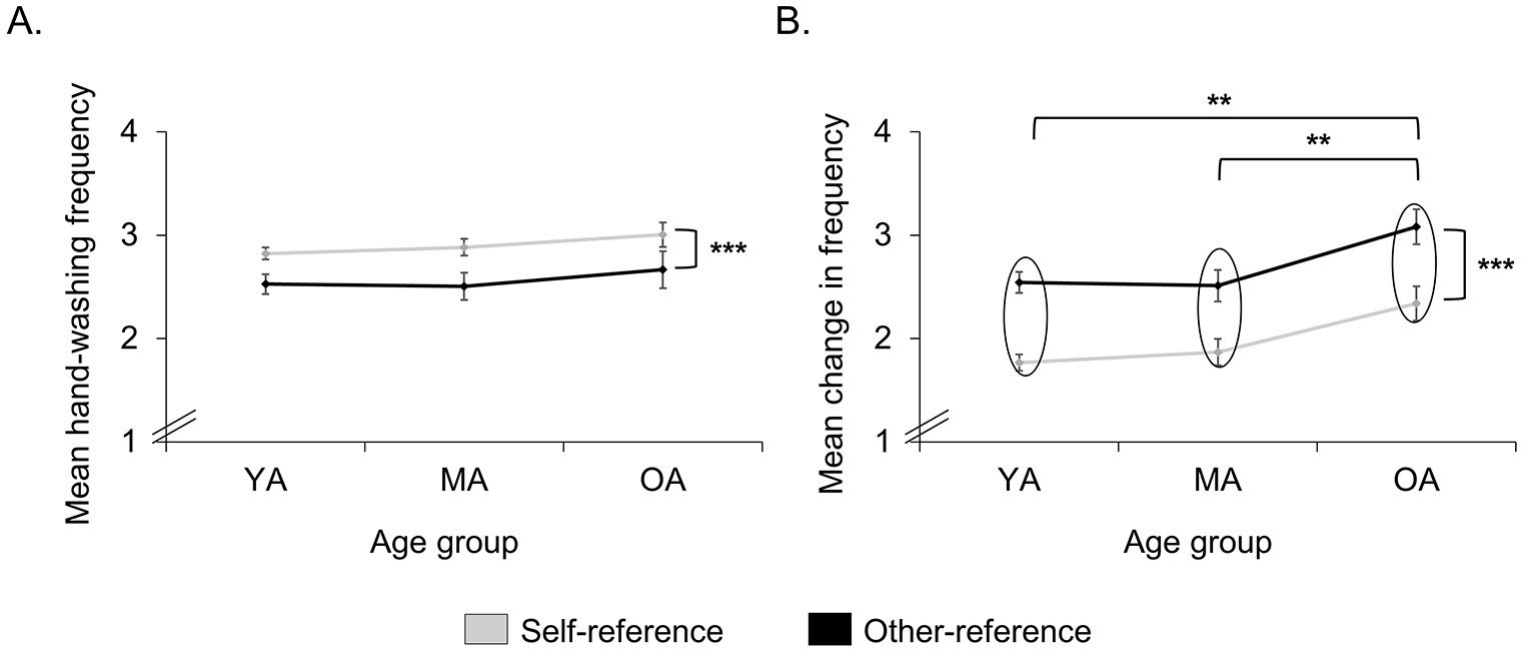
Mean Hand-Washing Frequency and Change *Note.* Mean hand-washing frequency (A.) and mean change in hand-washing frequency (B.) in situations with self-reference and other-reference for the three age groups (young age, middle age, and best/older age). Mean hand-washing frequency on a scale from “0 = never” to “4 = always” (A.) and mean change in hand-washing frequency on a scale from “0 = unchanged” to “4 = very much more” (B.). Error bars denote standard error of the mean. **p < .01. ***p < .001.

A repeated measures analysis of variance (ANOVA) for frequency of hand-washing with the factors *Age Group* (YA, MA, OA) and *Situation* (self-reference, other-reference) showed a main effect for *Situation*, with participants reporting more frequent hand-washing in situations with self-reference as compared to situations with other-reference, *F*(1, 281) = 18.50, *p* < .001, $\eta_{p}^{2}$ = .062. There was no significant main effect of *Age Group* (*p* = .474) and no significant interaction (*p* = .879; see Figure 2, Panel A).

A repeated measures ANOVA for change in hand-washing with the factors *Age Group* (YA, MA, OA) and *Situation* (self-reference, other-reference) also showed a main effect for *Situation*. Participants reported a greater change of hand-washing in situations with other-reference as compared to situations with self-reference, *F*(1, 281) = 67.37, *p* < .001, $\eta_{p}^{2}$ = .193. In addition, there was a significant main effect of *Age Group*, *F*(2, 281) = 6.24, *p* = .002, $\eta_{p}^{2}$ = .043. Post-hoc *t*-tests for independent samples revealed a greater change in the OA group as compared to the YA group (*p* = .001) and the MA group (*p* = .003). The YA and the MA groups were not significantly different (*p* = .788). There was no significant interaction of *Age Group* and *Situation* (*p* = .756; see Figure 2, Panel B).

Importantly, the univariate ANOVA for the baseline item (after using the bathroom) showed no significant effect of *Age Group* for either frequency of (*p* = .130) or change in (*p* = .834) hand-washing.

**Figure 3:**
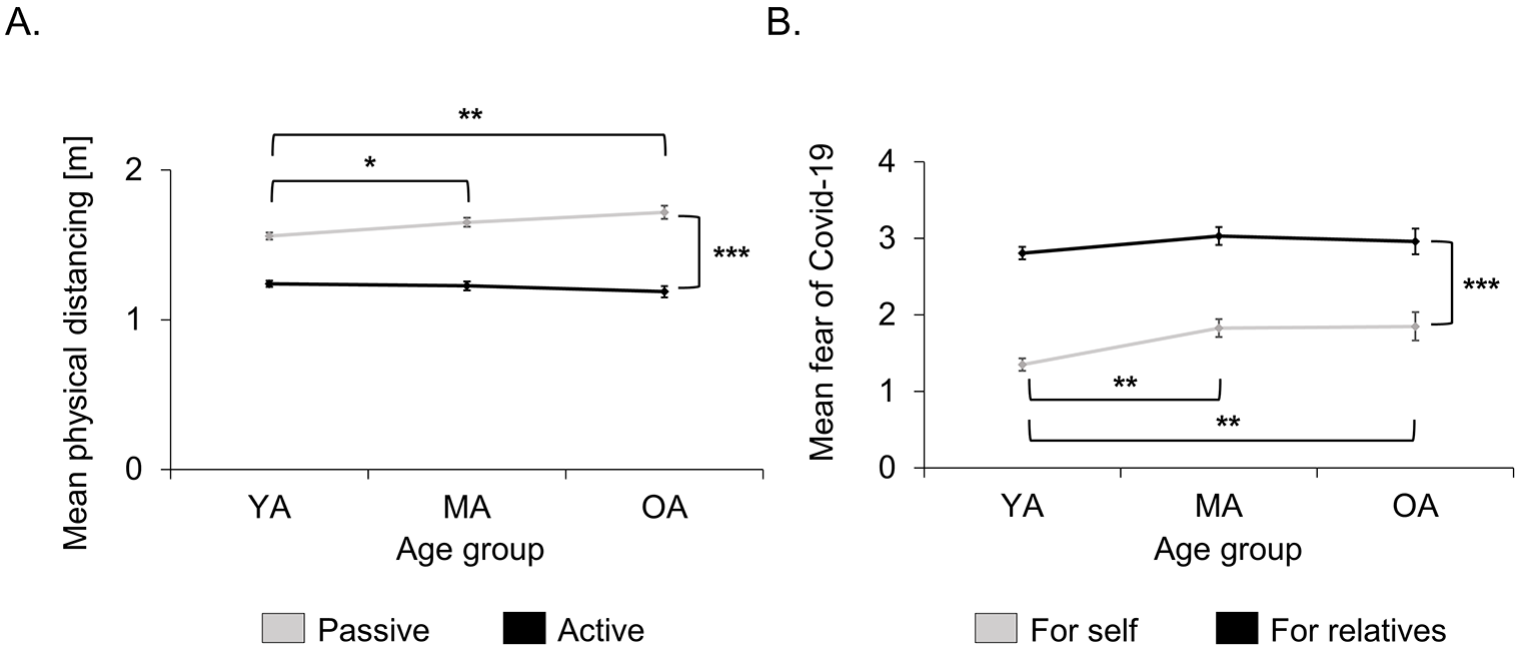
Mean Passive and Active Physical Distancing and Mean Fear of COVID-19 *Note.* Mean passive and active physical distancing (A.) and mean fear of COVID-19 for self and for relatives (B.) for the three age groups (young age, middle age, and best/older age). Mean fear of COVID-19 on a scale from “0 = not at all” to “4 = very much”. Error bars denote standard error of the mean. *p < .05. **p < .01. ***p < .001.

The repeated measures ANOVA for everyday physical distancing with the factors *Age Group* (YA, MA, OA) and *Distancing* (passive, active) showed a main effect for *Distancing*, *F*(1, 281) = 337.75, *p* < .001, $\eta_{p}^{2}$ = .546, with participants selecting greater physical distances in passive than in active distancing. There was no main effect of *Age Group* (*p* = .222). There was, however, a significant interaction of *Age Group* and *Distancing*, *F*(2, 281) = 7.28, *p* = .001, $\eta_{p}^{2}$ = .049. Post-hoc *t*-tests for independent samples showed significantly higher passive distancing in the OA group compared to the YA group (*p* = .001) but not to the MA group (*p* = .200), which also showed higher passive distancing than the YA group (*p* = .022). In contrast, there were no significant differences between the three groups for active everyday distancing (all *p*s > .2; see Figure 3, Panel A).

A repeated measures ANOVA for fear of COVID-19 with the factors *Age Group* (YA, MA, OA) and *Fear Target* (self, relatives) showed a main effect for *Fear Target*, with participants reporting more fear of COVID-19 for relatives than for themselves, *F*(1, 281) = 404.54, *p* < .001, $\eta_{p}^{2}$ = .590, and a main effect of *Age Group, F*(2, 281) = 4.61, *p* = .011, $\eta_{p}^{2}$ = .032, with the YA reporting less overall fear than the MA group (*p* = .007) and the OA group (*p* = .039). In addition, there was a significant interaction of *Age Group* and *Fear Target*, *F*(2, 281) = 3.32, *p* = .037, $\eta_{p}^{2}$ = .023. Post-hoc *t*-tests for independent samples showed significantly lower fear for themselves in the YA group compared to the MA group (*p* = .001) and the OA group (*p* = .005), which were not significantly different (*p* = .882). In contrast, there were no significant differences between the three groups for fear for relatives (all *p*s > .1; see Figure 3, Panel B).

General fear of COVID-19 was further investigated by calculating bivariate correlations with change in hand-washing frequency, physical distancing, and pathological hand-washing across all participants. Of note, there were significant associations of change in hand-washing frequency and passive physical distancing with both participants’ fear for themselves, *r*(282) = .19, *p* = .002 and *r*(282) = .19, *p* = .002, respectively, and for relatives, *r*(282) = .26, *p* < .001 and *r*(282) = .17, *p* = .005, respectively. Participants reporting higher fear levels also reported greater changes in hand-washing frequency and more passive physical distancing. In contrast, active physical distancing was not associated with general fear of COVID-19 (both *p*s > .08). In addition, general fear of COVID-19 for both themselves as well as for relatives was correlated with pathological hand-washing, *r*(282) = .22, *p* < .001 and *r*(282) = .20, *p* = .001, respectively. Participants reporting higher fear levels also reported washing their hands more frequently and longer than necessary (see Supplementary Materials for group-specific correlations).

Univariate ANOVAs with the factor *Age Group* (YA, MA, OA) yielded no age group differences with regard to the use of soap (*p* = .103) or disinfectant (*p* = .448), trying not to touch one’s face (*p* = .699), the average amount of people not belonging to one’s household met per day (*p* = .633), or pathological hand-washing (*p* = .248; see Table 1 for all means and standard deviations).

There was, however, a marginally significant main effect of *Age Group* for the subjective importance of observing the recommendations regarding hand-washing, *F*(2, 281) = 2.88, *p* = .058, $\eta_{p}^{2}$ = .020, with the OA group perceiving the observation of these recommendations as significantly more important than the YA group (*p* = .025) and the MA group (*p* = .032). In addition, there was a significant main effect of *Age Group* for attention to observing adequate physical distancing during interactions, *F*(2, 281) = 5.09, *p* = .007, $\eta_{p}^{2}$ = .035, with the OA group reporting significantly more attention than the YA group (*p* = .002) and also marginally more attention than the MA group (*p* = .076).

## Discussion

This survey provides some insight into how health behavior habits in different age groups recently changed based on the actual pandemic situation and current recommendations for the prevention of infections. Importantly, the survey shows an increase in hand-washing after situations carrying a direct risk of infection by others (touching another person or an object touched by other people). However, conditions might still not allow for consistent hand-washing in these situations as the overall hand-washing is still lower than after situations that do not involve direct contact with others. This should urgently be investigated in further surveys.

Importantly, overall change in hand-washing frequency was highest in the best/older age group, compared to both the young and the middle age group. It could thus be hypothesized that the best/older age group is well aware of their at-risk status and is therefore willing to change their behavior more strongly than the younger survey participants. Indeed, general fear of COVID-19 correlated positively with changes in hand-washing frequency and with passive physical distancing. In addition, the best/older age group reports a significantly higher fear of contracting COVID-19 than the younger age group. In contrast, the middle age group reports a comparable level of fear, but no corresponding change in behavior. However, when fear of contracting COVID-19 was included as a covariate, effect sizes decreased but the reported results still retained significance.

Previous research showed increased health behavior when the framing of the health message matched participants’ emotional states (Gerend & Maner, 2011). Given the uncertain situation and the emphasis on age as the main risk factor at the beginning of the pandemic, it is understandable that older participants were generally more scared than younger participants. The initial “loss-framed” campaigns focusing on the risk of insufficient hand-washing and physical distancing thus might have led to stronger behavior changes in this age group. Should the pandemic worsen again in the future, it might therefore be worthwhile to also focus on “gain-framed” campaigns for the younger age groups stressing the (societal) benefits of hand-washing and physical distancing. In addition, health behavior can be promoted by correcting misperceptions of injunctive norms (Reid & Aiken, 2013). It might therefore be helpful to provide self-tests of hand-washing frequency and physical distancing that allow people to compare their own perceptions of acceptable behavior to the parameters actually considered acceptable by a representative sample.

With regard to age group differences, the young age group is somewhat less consistent implementing physical distancing in real life. When confronted with a selection of varying physical distances in an interpersonal situation, 12% of survey participants aged 30 or younger chose a distance of only 1 meter to have a conversation. This percentage was significantly lower in both older age groups. However, all participants seem to struggle with enforcing an appropriate physical distance when their conversational partner is not following recommendations. About half of the younger participants (53%) would actively stop their conversational partner from approaching any further at a distance of only 1 meter, with this percentage rising in the middle age group (57%) to almost two thirds (65%) of the best/older age group. As this group is most at-risk for complications from COVID-19, clinical psychologists might be called upon to provide assistance by instructing the general public on socially acceptable assertive behavior (e.g. based on Hinsch & Pfingsten, 2007).

Clinical psychological research should also monitor the incidence of compulsive washing as seen in obsessive compulsive disorder (OCD). It seems worrisome that fear of contracting COVID-19 was associated with self-reported more frequent and longer hand-washing than necessary across all age groups in our sample. According to the classic model of OCD by Salkovskis (1985), the reduction of anxiety through neutralizing behavior (i.e. hand-washing) provides powerful negative reinforcement, thereby increasing the likelihood of its occurrence in the future. As the knowledge about OCD in the general public is still rather low (Coles, Heimberg, & Weiss, 2013), clinical psychologists should try to offer expert opinions on the chance of increasing rates of OCD in the wake of the pandemic whenever possible. On a related note, recommendations regarding physical and social distancing could be detrimental for people suffering from depressive disorders or social phobia. This should also be closely monitored in the future.

There are also several limitations: The sample in this survey is rather small, self-selected, and probably highly educated, with many participants reporting a high degree of formal schooling and almost 40% attending university at the time of data collection. It would therefore be worthwhile to investigate a larger and more representative sample. As our current sample was too small for meaningful analyses with regard to gender, it would be especially informative for future surveys to examine how the general recommendations are perceived and implemented in men as compared to women and if this changes with increasing age. Unfortunately, we did not inquire whether participants were experiencing COVID-19 symptoms at the time of taking the survey. Future surveys should include this question to allow for more in-depth analyses. In addition, the observed findings were quite likely heavily influenced by the time period of data collection as infections were rising quickly and it was uncertain if and how the epidemic could be controlled in Germany at the time. While it is important to have assessed the data for this period in the pandemic, it would be worthwhile to revisit the survey questions at present (after many of the restrictions have been lifted) and examine if the behavioral changes reported earlier are still being maintained. In addition, results might be specific for Germany, as government reactions to the pandemic differed in different countries. It would therefore be informative to gather and compare similar data from other countries.

Overall, it has to be noted that all age groups rate their observance of recommendations regarding hand-washing and physical distancing as very important and that the use of soap during hand-washing was very high in this sample, suggesting a good knowledge and acceptance of the current recommendations (Bundeszentrale für gesundheitliche Aufklärung, 2020). A sharp decrease on this year’s influenza rates also testify to the effectivity of the current overall measures with regard to physical distancing (Buchholz, Buda, & Prahm, 2020). Our results furthermore show that recommendations given in a pandemic situation can in fact break through relevant habits. Whether this effect is mainly based on reflective decision-making (e.g. salient recommendation) or on impulsive processes (e.g. actual fear) should be further investigated. An additional challenge is now the long-term maintenance of these new adaptive behaviors as well as the management of potential negative effects of physical distancing and increased hand-washing on mental health.

## Supplementary Materials

The Supplementary Materials contain the English translation of the items analyzed in the manuscript (the original items are available from the authors upon request) and group-specific correlations and *p*-values for fear and age group (for access see Index of Supplementary Materials below).

10.23668/psycharchives.4558 Supplement 1 Supplementary materials to "Widespread recommendations can change our habits of hand-washing and physical distance during the COVID-19 pandemic" [Additional information].

BiehlS. C.
SchmidmeierM.
WechslerT. F.
KroczekL. O. H.
MühlbergerA.
 (2021). Supplementary materials to "Widespread recommendations can change our habits of hand-washing and physical distance during the COVID-19 pandemic"
[Additional information]. PsychOpen. 10.23668/psycharchives.4558PMC966712536397781
