# Supplementary material for: Widespread Recommendations Can Change Our Habits of Hand-Washing and Physical Distance During the COVID-19 Pandemic
Source: Clin Psychol Eur. 2021 Mar 10;3(1):e3061. doi: 10.32872/cpe.3061 (PMC9667125; doi:10.32872/cpe.3061)
Supplement: Supplement 1 [file cpe-03-3061-s1.pdf]

Supplement 1 – English translation of the items analyzed in the manuscript[1]

Question	Scale
How frequently do you currently wash your hands [after using the bathroom, before eating, after entering your flat/ house, after blowing your nose, after coughing/ sneezing in your hand, after touching another person not living in the same household, after touching an object that is also touched by other people]?	“0 = never” to “4 = always”
How frequently do you wash your hands in this situation [above], compared to the past?	“0 = unchanged” to “4 = very much more”
Do you use soap when washing your hands?	“0 = never” to “4 = always”
Do you use disinfectant to clean your hands?	“0 = never” to “4 = always”
Do you try not to touch your face, if possible?	“0 = never” to “4 = always”
Do you pay attention to observing the recommended physical distance to other people?	“0 = never” to “4 = always”
How many people who are not living in your household do you roughly meet per day?	Open response format
In your opinion, how important is it to mind good hand hygiene for yourself right now?	“0 = not at all” to “4 = very much”
How scared are you that you might fall ill with the virus?	“0 = not at all” to “4 = very much”
How scared are you that a relative might fall ill with the virus?	“0 = not at all” to “4 = very much”
I am washing my hands more frequently and longer than necessary.	“0 = not at all” to “4 = very much”
Which distance would you currently be most comfortable with during a conversation?	3 pictures (1m, 1.5m, 2m)
When would you stop someone who is approaching you to talk to you?	3 pictures (1m, 1.5m, 2m)

[1] The original items are available from the authors upon request.

Supplement 2 – Group-specific correlations and *p*-values

**Age group**	**Fear for themselves**	***r***	***p***
Young age	Change in hand-washing frequency	**.18**	**.032**
	Active physical distancing	-.07	.389
	Passive physical distancing	.15	.072
	Pathological hand-washing	**.21**	**.008**
Middle age	Change in hand-washing frequency	**.25**	**.019**
	Active physical distancing	-.004	.971
	Passive physical distancing	.13	.242
	Pathological hand-washing	**.24**	**.027**
Best/ older age	Change in hand-washing frequency	-.003	.986
	Active physical distancing	**-.29**	**.044**
	Passive physical distancing	.19	.197
	Pathological hand-washing	**.31**	**.035**

**Age group**	**Fear for relatives**	***r***	***p***
Young age	Change in hand-washing frequency	**.30**	**>.001**
	Active physical distancing	-.01	.867
	Passive physical distancing	.15	.060
	Pathological hand-washing	.16	.055
Middle age	Change in hand-washing frequency	**.37**	**<.001**
	Active physical distancing	-.01	.948
	Passive physical distancing	.16	.143
	Pathological hand-washing	**.29**	**.007**
Best/ older age	Change in hand-washing frequency	-.08	.583
	Active physical distancing	-.25	.088
	Passive physical distancing	.14	.327
	Pathological hand-washing	.24	.102
